# Efficient Removal of Bisphenol A Using Nitrogen-Doped Graphene-Like Plates from Green Petroleum Coke

**DOI:** 10.3390/molecules25153543

**Published:** 2020-08-03

**Authors:** Zhipeng Liu, Quanyong Wang, Bei Zhang, Tao Wu, Yujiang Li

**Affiliations:** 1 Shandong Provincial Research Center for Water Pollution Control, School of Environmental Science and Engineering, Shandong University, Jinan 250100, China; 201812787@mail.sdu.edu.cn (Z.L.); zhangbeisdu@163.com (B.Z.); 2 China Urban Construction Design and Research Institute Co., Ltd., Jinan 250101, China; hkyyswang@163.com; 3 Key Laboratory of Colloid and Interface Science of Education Ministry, Shandong University, Jinan 250100, China

**Keywords:** green petroleum coke, nitrogen doping, bisphenol A, electrostatic attraction, adsorption

## Abstract

Green petroleum coke, a form of industrial waste produced in the oil-refining process, was used to synthesize nitrogen-doped graphene-like plates (N-GLPs) together with melamine. In this study, characterization and batch experiments were performed to elucidate the interaction mechanism of N-GLPs and bisphenol A (BPA). Structural analysis of N-GLPs, including scanning electron microscopy (SEM), X-ray diffraction (XRD), Fourier transform infrared spectroscopy (FT-IR), Brunauer-Emmett-Teller (BET), and X-ray photoelectron spectroscopy (XPS), showed an obvious graphene-like structure and successful nitrogen doping. In addition, compared with 8.0 m^2^/g for green petroleum coke, the BET surface area of N-GLPs markedly increased to 96.6 m^2^/g. The influences of various factors, including contact time, temperature, and initial pH on BPA removal efficiency were investigated. It was found that 92.0% of BPA was successfully removed by N-GLPs at 50 °C. Based on the adsorption experiments, it was shown that electrostatic attraction, hydrogen bonding, and π-π interaction enhanced the adsorption capacity of N-GLPs for BPA. According to the thermodynamic data, the adsorption process was spontaneous, physical, and endothermic in nature. Therefore, N-GLPs are efficient adsorbent material to remove BPA from wastewater.

## 1. Introduction

As a phenolic compound with high production, bisphenol A (BPA) is widely utilized in the manufacturing of epoxy resins and polycarbonate plastics [1,2]. Due to its pervasive application, a tremendous amount of BPA is discharged into the environment. Indeed, BPA has been confirmed to be present in a broad variety of areas, such as water systems, air, sediment, landfills, and some food matrices [3]. BPA is known as an endocrine-disrupting compound and may lead to deleterious effects on humans due to its estrogenic activity [4]. As the existence of BPA in water environments constitutes a potential major hazard for both humans and aquatic organisms, it is critical to develop an efficient method to remove BPA.

Among the various methods available for the removal of BPA, adsorption has attracted great attention due to its economic applicability, high efficiency, easy operation, and hypotoxicity [5,6]. The particular adsorbent used in an adsorption process determines both adsorption efficiency and cost and thus plays a key role in its use and effectiveness. Carbonaceous materials, including activated carbon, graphene, graphene oxide, and reduced graphene oxide, have been widely utilized in water purification processes. They can achieve rapid adsorption rates due to their large surface area and pore volume [7]. However, these adsorbents possess numerous disadvantages, such as high production cost, low productivity, and challenging regeneration. All of these disadvantages have demonstrated the necessity to identify or develop a novel adsorbent with superior adsorption efficiency and lower production cost to remove BPA from wastewater.

Green petroleum coke is a by-product from the carbonization of petroleum feedstock and oil-refining process [8,9]. It offers numerous advantages, such as high fixed carbon content, low content of ash, extensive sources, low cost, and abundant benzene rings or aromatic domains [10,11]. However, as a by-product with high production, a vast amount of green petroleum coke is not utilized in a timely manner and is instead stacked. This presents a major environmental concern because soluble substances in green petroleum coke will permeate into the groundwater and soil during the leaching process [12]. Consequently, the development of a method to promptly and effectively utilize green petroleum coke is needed. Considering its major advantages, it is feasible for green petroleum coke to serve as a raw adsorbent material to deal with BPA in aqueous solution.

In this work, we report a cost-effective method for synthesizing nitrogen-doped graphene-like plates from green petroleum coke as a novel adsorption material. Green petroleum coke was calcined at high temperature as a post-treatment to obtain petroleum coke as a precursor. Melamine was chosen as the nitrogen source for the nitrogen-doped graphene-like plates (N-GLPs). Nitrogen doping on a carbon network has been confirmed to introduce positive charge and then augment electrostatic interaction [13]. In addition, nitrogen doping on petroleum coke would obtain pyridinic N, pyrrolic N, and graphitic N, thus regularizing the crystal structure [14]. Moreover, a π-π interaction might form between nitrogen-doped graphene-like plates and benzene rings in BPA. Scanning electron microscopy (SEM), energy-dispersive X-ray spectroscopy (EDX), X-ray diffraction (XRD), Fourier transform infrared spectroscopy (FT-IR), the Brunauer-Emmett-Teller (BET) specific surface area, and X-ray photoelectron spectroscopy (XPS) were utilized to analyze the physicochemical properties of nitrogen-doped graphene-like plates. Subsequently, the influence of adsorption conditions, including contact time, temperature, and pH, were investigated, thereby determining the optimum conditions for maximum removal efficiency of BPA. The adsorption mechanism of BPA was also elucidated with the structure of nitrogen-doped graphene-like plates and the adsorption isotherms.

## 2. Results and Discussions

### 2.1. Optimization of Adsorption Materials

#### 2.1.1. Effect of N-Doped Temperature

N-doped temperature is a key factor affecting a material’s physicochemical properties. For the variation in physicochemical properties, we used several techniques, e.g., SEM, XED, FT-IR, BET, and XPS, to characterize the obtained materials. In this work, we fixed the N-doping time at 2 h and varied the N-doped temperature to 350, 550, and 750 °C to produce three N-doped samples. After calcination and nitrogen doping, green petroleum coke particles transformed into N-GLPs. We defined the three obtained N-doped samples as N-GLPs 350_2_, N-GLPs 550_2_, and N-GLPs 750_2_.

##### SEM

**Figure 1:**
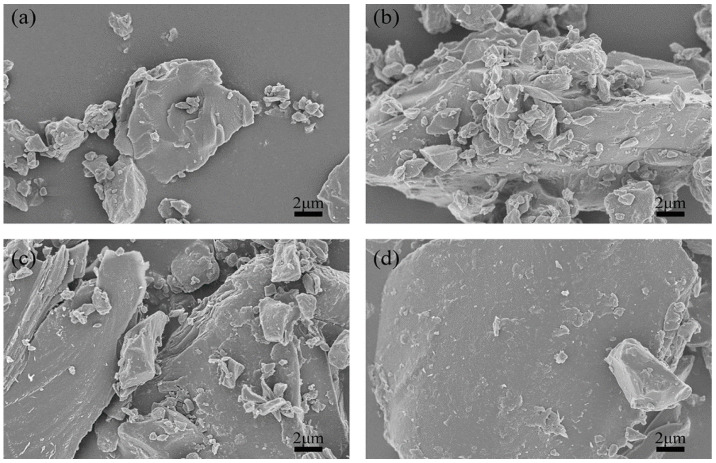
Effect of N-doped temperature on material’s optimization: (**a**) green petroleum coke, (**b**) nitrogen-doped graphene-like plates (N-GLPs) 350_2_, (**c**) N-GLPs 550_2_, and (**d**) N-GLPs 750_2_.

SEM was performed to analyze the morphologies and microstructures of the green petroleum coke and N-GLP samples obtained from calcination at 350, 550, and 750 °C. SEM images of green petroleum coke, N-GLPs 350_2_, N-GLPs 550_2_, and N-GLPs 750_2_ composites at the same magnification are shown in Figure 1. Compared with green petroleum coke’s irregular granular structure (Figure 1a), N-GLP samples with N-doping and thermal processing (Figure 1b–d) showed a more regular and larger lamella structure. The SEM images indicated that calcination and nitrogen doping transformed the green petroleum coke into a more regular and neater lamella structure. Moreover, the SEM image of N-GLPs 750_2_ (Figure 1d) demonstrated that N-doping at 750 °C obtained the N-GLP sample with the smoothest lamellar structure among the samples mentioned above.

##### XRD

**Figure 2:**
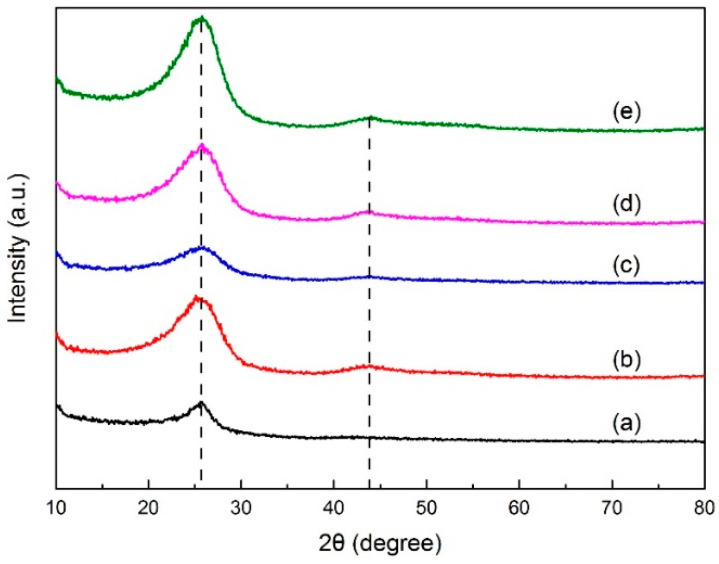
X-ray diffraction (XRD) patterns of (a) green petroleum coke, (b) graphene, (c) N-GLPs 350_2_, (d) N-GLPs 550_2_, and (e) N-GLPs 750_2_.

X-ray diffraction analysis determined the crystal structure in green petroleum coke and N-GLP samples by measuring the reflection angle from the crystal lattice. The XRD profiles of green petroleum coke, graphene, N-GLPs 350_2_, N-GLPs 550_2_, and N-GLPs 750_2_ are given in Figure 2. As shown in Figure 2, green petroleum coke, graphene, and all of the N-GLP samples exhibited a characteristic peak at 2θ = 26° on their XRD diffraction spectrum. With the temperature increasing, a small peak at 2θ = 44° on XRD diffraction spectrums of N-GLP samples could be identified. The peak at 2θ = 26° and 2θ = 44° corresponded to crystalline regions of diffraction from layering of graphitic carbons and formation of small 2D lattices, respectively [15]. In addition, the peak height and intensity at 2θ = 26° of the N-GLP samples were higher than those of green petroleum coke, which demonstrated that green petroleum coke was led to an orderly crystal structure through calcination and N-doping. Compared with other samples, N-GLPs 750_2_ (Figure 2e) exhibited the highest peak intensity. The XRD results revealed that the green petroleum coke tended to be graphitized to a greater extent with N-doping and the increase in temperature [16].

##### FT-IR

**Figure 3:**
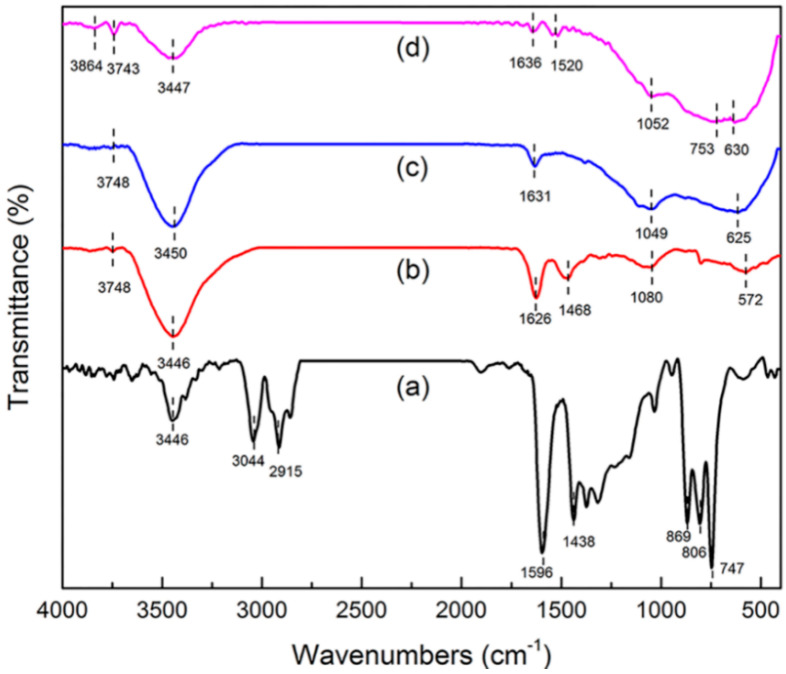
Fourier transform infrared spectroscopy (FT-IR) spectra of (a) green petroleum coke, (b) N-GLPs 350_2_, (c) N-GLPs 550_2_, and (d) N-GLPs 750_2_.

Information about surface functional groups and chemical bonding behavior was obtained by FT-IR [17,18]. The FT-IR spectra of green petroleum coke, N-GLPs 350_2_, N-GLPs 550_2_, and N-GLPs 750_2_ are shown in Figure 3. For green petroleum coke (Figure 3a), the bands in the FT-IR spectrum can be summarized as follows: (1) the typical band at 3446 cm^−1^ can be attributed to -OH stretching vibration; (2) the bands at 3044 and 2915 cm^−1^ can be assigned to C-H asymmetric and symmetric stretching vibrations of CH_2_ and CH_3_ groups [19]; (3) the bands at 1596 and 1438 cm^−1^ can be identified as C-C stretching vibration; and (4) the bands at 869, 806, and 747 cm^−1^ can be ascribed to the characteristic peaks of aromatic structure [20,21]. The characteristic bands of green petroleum coke decreased apparently with increasing N-doping temperature. In addition, the bands at 3044 and 2915 cm^−1^ disappeared, indicating that calcination removed some aromatic hydrocarbons with small molecular weight from the green petroleum coke. Compared with green petroleum coke, the FT-IR spectrum of N-GLPs exhibited a typical transformation process of bands with increasing N-doping temperature. For the FT-IR spectrum of N-GLPs 750_2_ (Figure 3d), five main regions were identified: (1) the bands at 3864 and 3743 cm^−1^ can be assigned to -OH stretching vibration [22]; (2) the band at 3447 cm^−1^ can be attributed to N-H symmetric stretching vibration [23]; (3) the band at 1636 cm^−1^ can be identified as N-H in-plane deformation vibration [23,24,25]; (4) the band at 1520 cm^−1^ can be ascribed to C=N stretching vibration, while the band at 1052 cm^−1^ can be assigned to C-N stretching vibration [25,26]; and (5) the bands at 753 and 630 cm^−1^ can be attributed to -NH_2_ vibration bands [27]. All of these characteristics indicated the existence of N-containing functional groups and the successful doping of nitrogen in N-GLP samples.

##### BET

It is important to measure specific surface area and pore volume of green petroleum coke and N-GLP samples in order to determine their physicochemical properties [28]. To identify the pore volume and specific surface area, we performed N_2_ sorption measurements. The BET method from the N_2_ adsorption isotherm was carried out to determine the BET surface area (S_BET_) of green petroleum coke and N-GLP samples, while the pore volume was obtained by holding the N_2_ volume at a relative pressure of P/P_0_ = 0.998. The S_BET_ and pore volume (V_p_) of the green petroleum coke and N-doped samples are listed in Table 1. The green petroleum coke only possessed a small S_BET_ and V_p_ of 8.0 m^2^/g and 0.013 cm^3^/g, respectively. After thermal processing and N-doping, the S_BET_ of N-GLPs 350_2_, N-GLPs 550_2_, and N-GLPs 750_2_ increased to 35.9, 44.8, and 65.4 m^2^/g, respectively. In addition, the V_p_ of N-GLPs 350_2_, N-GLPs 550_2_, and N-GLPs 750_2_ also increased to 0.029, 0.040, and 0.055 cm^3^/g, respectively. These analysis data indicated that N-GLP samples with a larger specific surface area and higher porosity would constitute potential adsorbents for BPA.

##### XPS

**Figure 4:**
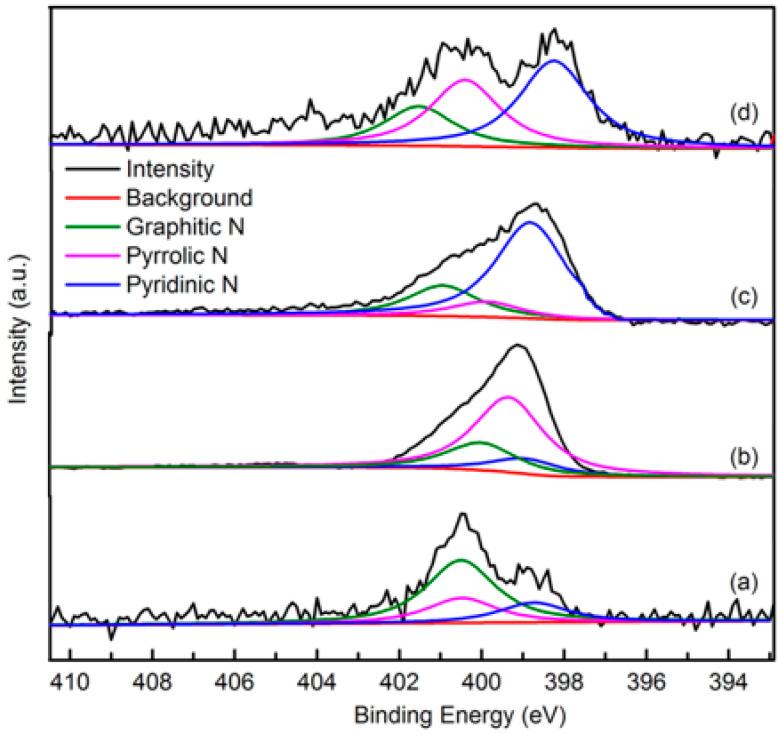
Narrow scanning N 1s X-ray photoelectron spectroscopy (XPS) spectra of (a) green petroleum coke, (b) N-GLPs 350_2_, (c) N-GLPs 550_2_, and (d) N-GLPs 750_2_.

The elemental composition and nitrogen bonding configurations in green petroleum coke and N-GLP samples were obtained by XPS characterizations. The detailed characteristics of the nitrogen species on the surface of the samples were probed by high-resolution N 1s spectra, as shown in Figure 4. For all samples, the raw peaks of N 1s were deconvoluted into three types of nitrogen species. The peaks around 398.8, 399.8, and 401.3 eV were ascribed to pyridinic N, pyrrolic N, and graphitic N, respectively [29,30,31]. The ratio of pyrrolic N:pyridinic N:graphitic N was 1:0.20:0.36 (overall 3.97 at%), 1:6.22:1.98 (overall 5.59 at%), and 1:2.20:1.68 (overall 6.21 at%) for N-GLPs 350_2_, N-GLPs 550_2_, and N-GLPs 750_2_, respectively. With increasing thermal annealing temperature, more graphitic N incorporated into the graphene-like plane due to superior thermal stability compared to pyrrole-like N [32,33,34,35]. Moreover, the binding energy of graphitic N increased with the increase in temperature, which was due to successful nitrogen doping [36,37,38]. The increase in graphitic N content indicate that N-GLPs can be successfully synthesized by calcining green petroleum coke with melamine. Moreover, nitrogen species and doping content can be controlled by synthesis conditions. Higher temperatures cause more nitrogen atoms to be doped into N-GLPs films, and graphitic N structures are formed preferably at higher temperatures [39]. Combining the nitrogen compositional species and nitrogen content, N-GLPs 750_2_ at 750 °C (Figure 4d) obtained the ideal N-doping effect.

Based on the analysis results of all characterizations, we chose 750 °C as the most appropriate N-doped temperature.

#### 2.1.2. Effect of N-Doping Time

N-doping time is another crucial factor affecting a material’s physicochemical properties, thereby further influencing a material’s adsorption capacities. In this study, we fixed N-doped temperature at 750 °C and varied the N-doping time from 2 to 6 h to produce three N-doped samples. The obtained three N-doping samples were defined as N-GLPs 750_2_, N-GLPs 750_4_, and N-GLPs 750_6_, corresponding to an N-doping time of 2, 4, and 6 h, respectively.

##### SEM

**Figure 5:**
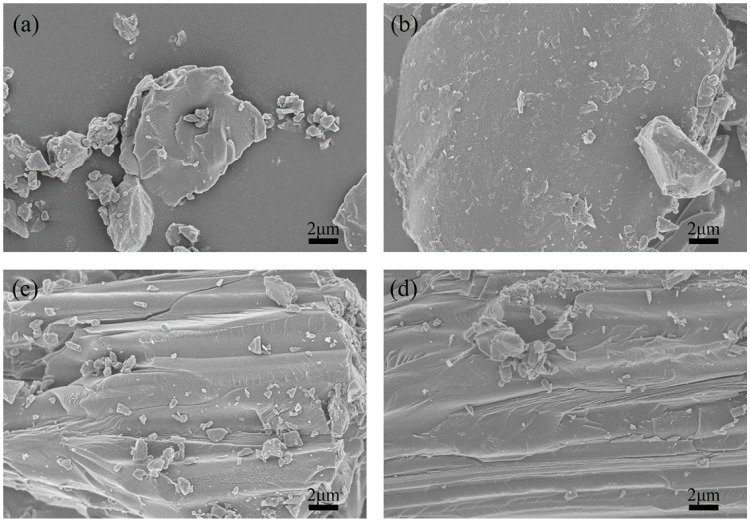
Effect of N-doping time on material’s optimization: (**a**) green petroleum coke, (**b**) N-GLPs 750_2_, (**c**) N-GLPs 750_4_, and (**d**) N-GLPs 750_6_.

The morphological and structural features of green petroleum coke and N-GLP samples were characterized by SEM. SEM images of green petroleum coke, N-GLPs 750_2_, N-GLPs 750_4_, and N-GLPs 750_6_ composites are shown in Figure 5. All N-GLP samples obtained at 750 °C (Figure 5b–d) exhibited a regular lamella structure in contrast to that of green petroleum coke (Figure 5a). Furthermore, the lamella structure became more apparent with the increase in N-doping time. As shown in Figure 5d, the lamella of N-GLPs 750_6_ was extremely obvious and defined, demonstrating that the structure of N-GLPs would become clear and ordered with longer N-doping time. Consequently, we identified that controlling the N-doping time at 6 h could obtain the N-GLP sample with the optimal lamella structure.

##### XRD

**Figure 6:**
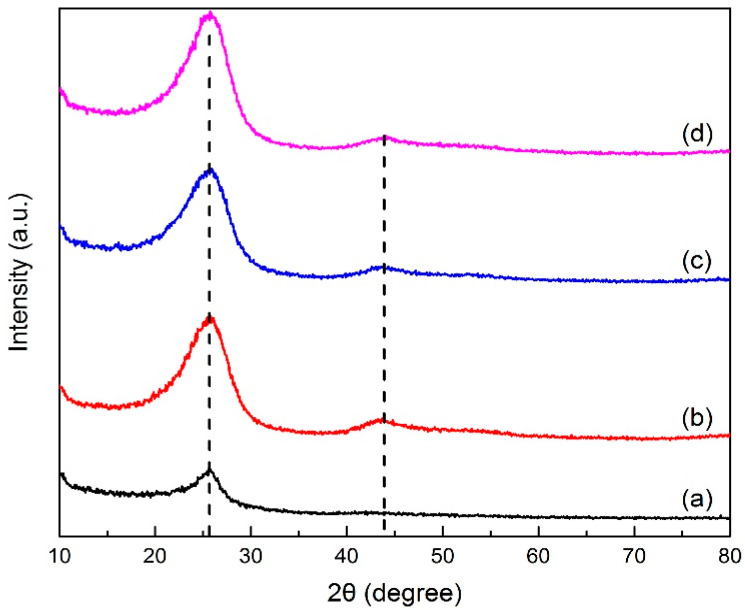
XRD patterns of (a) green petroleum coke, (b) N-GLPs 750_2_, (c) N-GLPs 750_4_, and (d) N-GLPs 750_6_.

The X-ray diffraction profiles of green petroleum coke, N-GLPs 750_2_, N-GLPs 750_4_, and N-GLPs 750_6_ are given in Figure 6. All the XRD diffraction spectrum of N-GLP samples showed two characteristic peaks at 2θ = 26° and 2θ = 44°, indicating that the crystal structures of N-GLP samples were identical to that of graphene. In addition, all of the N-GLP samples at 750 °C exhibited great peak height and intensity with narrow difference (Figure 6b–d). Compared with other N-GLP samples, N-GLPs 750_6_ (Figure 6d) revealed a slightly higher peak intensity. In this case, we concluded that N-GLP samples obtained at 750 °C contained an excellent crystal structure and increasing the N-doping time would narrowly improve the material property.

##### FT-IR

**Figure 7:**
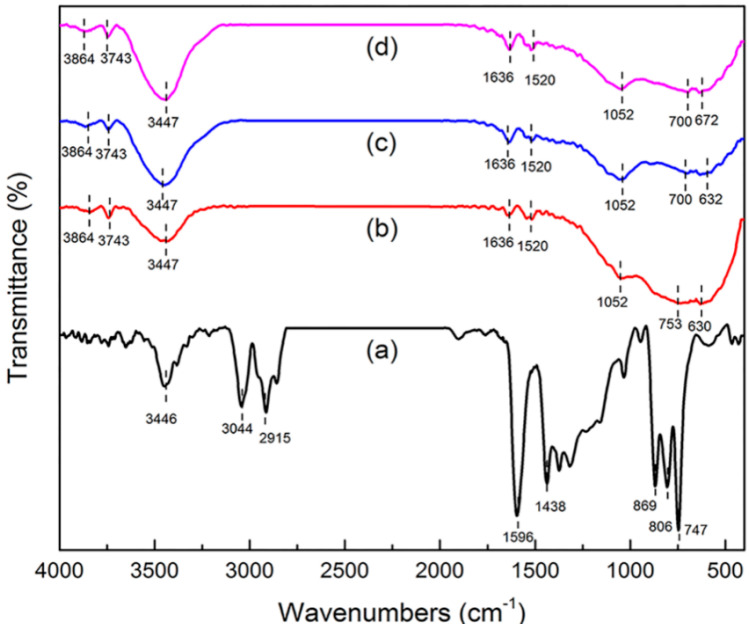
FT-IR spectra of (a) green petroleum coke, (b) N-GLPs 750_2_, (c) N-GLPs 750_4_, and (d) N-GLPs 750_6_.

The FT-IR spectra of green petroleum coke, N-GLPs 750_2_, N-GLPs 750_4_, and N-GLPs 750_6_ are shown in Figure 7. The N-GLP samples at 750 °C with different N-doping times exhibited similar characteristic bands, while the N-GLPs 750_4_ and N-GLPs 750_6_ (Figure 7c,d) showed some differences. Compared with N-GLPs 750_2_ (Figure 7b), for the FT-IR spectrum of N-GLPs 750_4_ (Figure 7c), two main differences could be identified: (1) the broad band at 753 and 630 cm^−1^ of N-GLPs 750_2_ shifted to 700 and 632 cm^−1^ with increasing N-doping time, and the intensity of these two bands was also lower, which indicated the decrease in -NH_2_ bands; and (2) the intensity of the band at 3447 cm^−1^ was higher, suggesting the improvement of N-H concentration. The FT-IR spectra also revealed that the increase in N-doping time would decrease unstable -NH_2_ bands and increase the concentration of stable N-H bands.

##### BET

The S_BET_ and V_p_ of the green petroleum coke and N-doped samples of different N-doping times are listed in Table 2. After extending the N-doping time, the S_BET_ of N-GLPs 750_2_, N-GLPs 750_4_, and N-GLPs 750_6_ increased to 65.4, 76.9, and 96.6 m^2^/g, respectively. The V_p_ of N-GLPs 750_2_, N-GLPs 750_4_, and N-GLPs 750_6_ also rose to 0.055, 0.068, and 0.082 cm^3^/g, respectively. Compared with green petroleum coke’s low S_BET_ and V_p_ parameters, N-GLPs 750_6_ calcined at 750 °C for 6 h could obtain larger S_BET_ and V_p_ and thus offer great adsorption efficiency.

##### XPS

**Figure 8:**
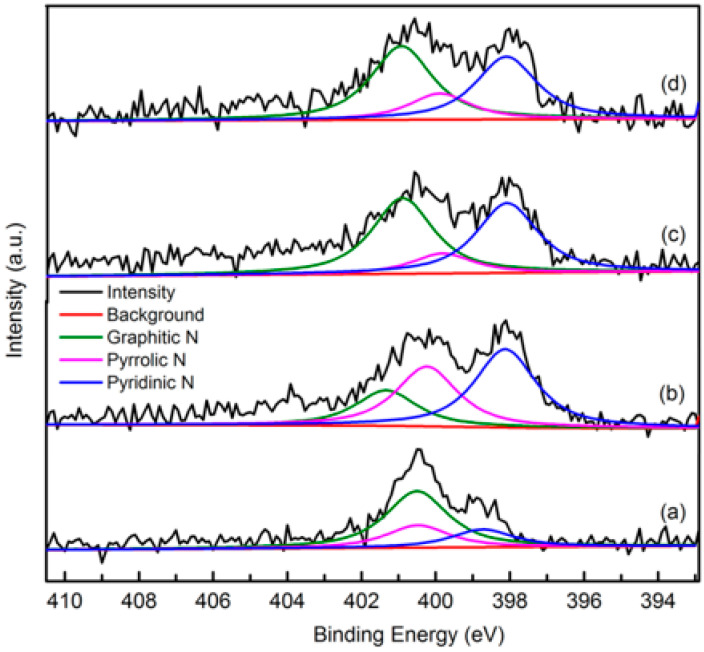
Narrow scanning N 1s XPS spectra of (a) green petroleum coke, (b) N-GLPs 750_2_, (c) N-GLPs 750_4_, and (d) N-GLPs 750_6_.

The N 1s XPS spectrum of green petroleum coke and N-GLP samples at different N-doping times is illustrated in Figure 8. The ratio of pyrrolic N:pyridinic N:graphitic N was 1:2.20:1.68 (overall 6.21 at%), 1:3.51:3.79 (overall 6.37 at%), and 1:2.40:2.81 (overall 7.11 at%) for N-GLPs 750_2_, N-GLPs 750_4_, and N-GLPs 750_6_, respectively. With increasing N-doping time, the total content of doped nitrogen for N-GLP samples rose. In addition, the increase in graphitic N indicated that N-GLP samples at longer N-doping time would become more stable. N-GLPs 750_6_ (Figure 8d) contained the greatest nitrogen content and a stable nitrogen composition, thereby having the potential to improve the adsorption capacity.

Combining the analysis results of all characterizations, we chose 6 h as the optimal N-doping time. Therefore, N-GLPs 750_6_ at 750 °C for 6 h was the ideal adsorbent used in further adsorption experiments.

### 2.2. Adsorption of BPA on N-GLPs 750_6_

#### 2.2.1. Effect of Contact Time and Initial BPA Concentration

**Figure 9:**
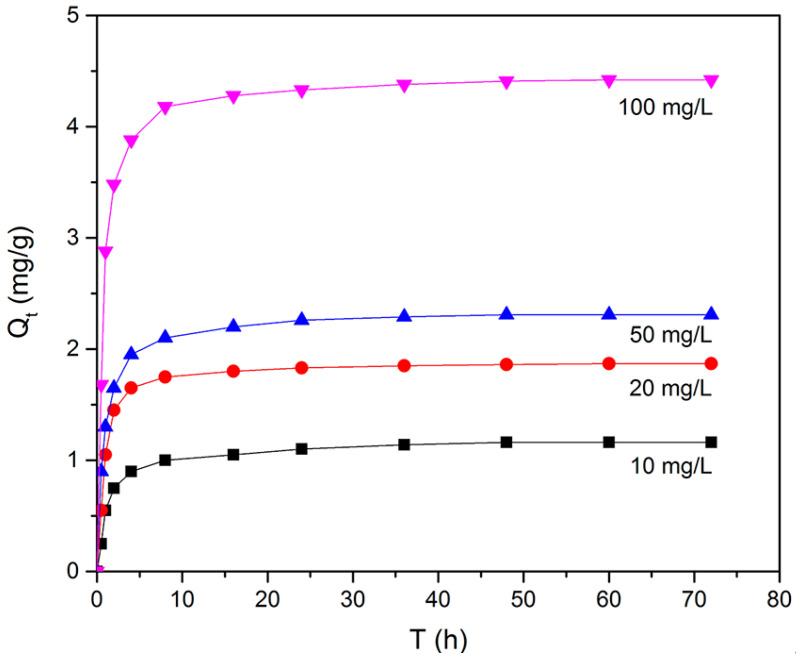
Effect of initial concentration and contact time on removal efficiency (experimental conditions: adsorbent dose = 7.0 g/L, initial concentration = 10–100 mg/L, pH = 6, T = 30 °C).

The effect of contact time on the adsorption of BPA at various BPA concentrations was investigated at 30 °C. As shown in Figure 9, the adsorption capacity of N-GLPs 750_6_ rose with increasing initial BPA concentration. Moreover, with increasing contact time, it was observed that the adsorption capacity of N-GLPs 750_6_ rose rapidly in the first 10 h and then slowly attained the maximum adsorption capacity after 48 h, indicating the adsorption equilibrium. Due to the large molecular size of BPA, it took a long time for BPA molecules to move to the surface of N-GLPs 750_6_ through Brownian movement. BPA then entered the adsorbent and was adsorbed under various forces, thus enhancing the adsorption time [40,41]. In further adsorption studies, based on the above results, 48 h was selected as the most appropriate contact time to reach the adsorption equilibrium. Pseudo-first-order and pseudo-second-order kinetic models were adopted to understand the adsorption behavior of N-GLPs 750_6_ for BPA [42,43,44]. As shown in Table S1 (Supplementary Materials), the adsorption kinetics of BPA on N-GLPs 750_6_ could be better fitted by the pseudo-second-order model (0.9993–1) than the pseudo-first-order model (0.8343–0.9152), indicating that the adsorption process was controlled by multiple factors. When the adsorption was far from equilibrium, the adsorption process was governed by the rate of surface reactions (electrostatic interaction and π-π interaction), and the intraparticle diffusion became predominant before approaching equilibrium [45,46].

#### 2.2.2. Effect of pH

**Figure 10:**
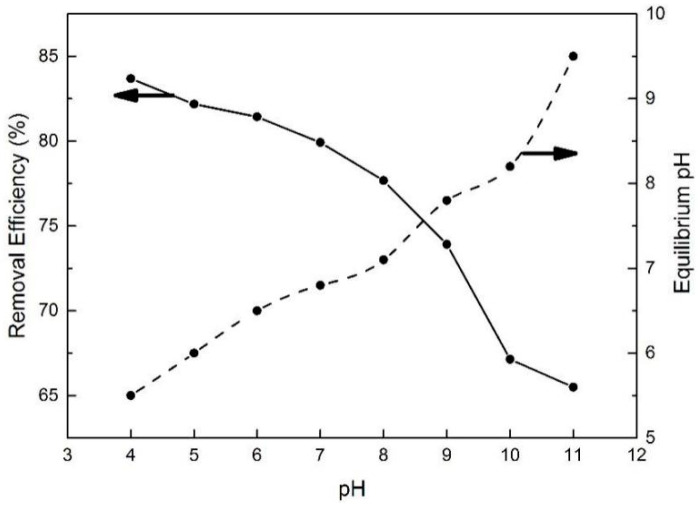
Effect of pH on removal efficiency and equilibrium pH (experimental conditions: initial concentration = 10 mg/L, adsorbent dose = 7.0 g/L, contact time = 48 h, T = 30 °C).

**Figure 11:**
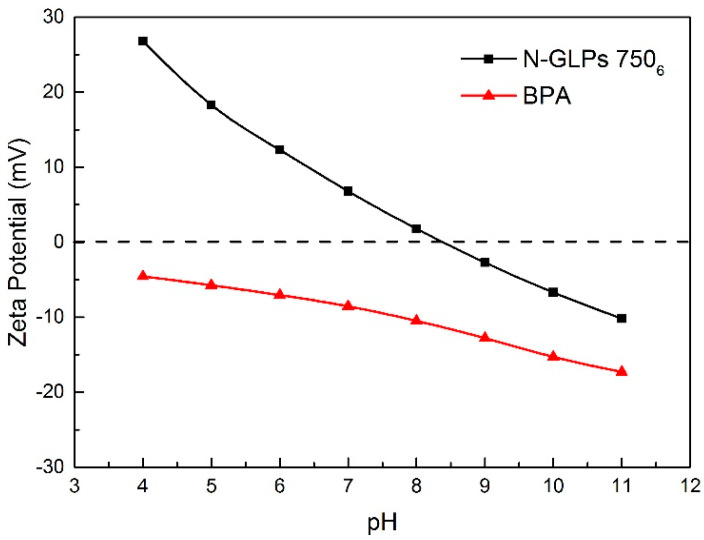
Zeta potentials of adsorbent and pollutant in 0.001 M NaCl solution (25 °C).

As one of the most important adsorption parameters for practical water environmental remediation, the initial pH of a solution intensively influences the surface physicochemical properties of adsorbents [46]. As shown in Figure 10, the initial pH of the BPA aqueous solution was varied from 4 to 11 to investigate its effect on BPA removal efficiency. With the initial pH increasing to 8, the removal efficiency of BPA on N-GLPs 750_6_ decreased slightly. Then, the removal efficiency decreased rapidly as the pH further increased. This phenomenon can be attributed to the following: (1) The charge of the adsorbent and pollutant. To determine the conditions of charge on N-GLPs 750_6_ layers and BPA aqueous solution, zeta potential was measured. The zeta potential values of N-GLPs 750_6_ dispersion and BPA solution were measured at 25 °C in 10^−3^ mol/L NaCl solution, and the results are presented in Figure 11. A charge inversion from positive to negative was observed in the N-GLPs 750_6_ dispersion with the BPA solution containing a negative charge. At pH = 4–6, the N-GLPs 750_6_ layers contained high positive charge density. Therefore, strong electrostatic attraction existed between N-GLPs 750_6_ and BPA. Correspondingly, the removal efficiency of BPA showed a high level at pH = 4–6. When the positively charged N-GLPs 750_6_ interacted with electronegative pollutants, it possessed enhanced electrostatic attraction for removing BPA in aqueous solution. With the pH further increasing, there were more negatively charged sites on the surface of adsorbents because of the available hydroxyl ions [47]. In this way, electrostatic repulsion between the negatively charged BPA and N-GLPs 750_6_ decreased the adsorption capacity. (2) The pKa of BPA (9.5), which also affected removal efficiency [48]. When the pH was less than the pKa, BPA was present in its molecular form, thus containing a lower negative charge. However, when the pH of the solution was higher than the pKa, BPA was present in the form of the phenolate ion. With the hydroxyl ion increasing, BPA was highly negatively charged. Moreover, the number of negatively charged sites on the N-GLPs 750_6_ also rose. The electrostatic repulsion between BPA and N-GLPs 750_6_ induced the decrease in adsorption capacity and removal efficiency. The equilibrium pH for BPA adsorption was also measured. As shown in Figure 10, when the equilibrium pH was lower, the removal efficiency of BPA increased. In contrast, when the equilibrium pH was higher, the adsorption of BPA was negatively influenced, which corresponds to the conclusions from the analysis of the initial pH. Combining the removal efficiency and the feasibility in practical application, we chose pH of 6 as the optimal adsorption condition.

#### 2.2.3. Effect of Temperature

**Figure 12:**
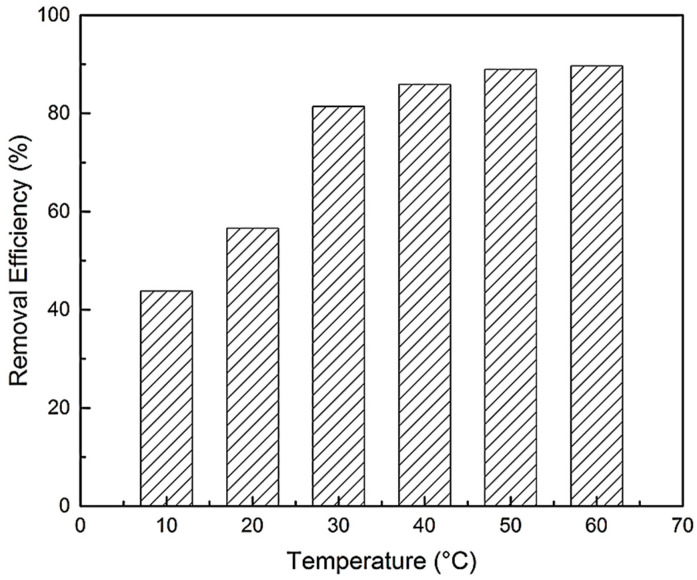
Effect of temperature on removal efficiency (experimental conditions: adsorbent dose = 7.0 g/L, initial concentration = 10 mg/L, contact time = 48 h, pH = 6.0).

Temperature exerts a major effect on the adsorption capacity of BPA. The effect of temperature on the adsorption of BPA with N-GLPs 750_6_ was investigated in a temperature range from 10 to 60 °C at constant pH (6.0) and contact time (48 h). As shown in Figure 12, the removal efficiency of BPA on N-GLPs 750_6_ rose with increasing temperature, indicating that the adsorption of BPA onto N-GLPs 750_6_ was endothermic. In addition, Brownian movement of BPA molecules increased with rising temperature, which resulted in an increase in the rate of mass transfer from the liquid phase to the solid/liquid phase [49]. BPA would have been attached on the surface of N-GLPs 750_6_ and led to further interaction when they became closer. The viscosity of bulk also decreased with increasing temperature, thus augmenting the mobility of BPA in aqueous solution. Moreover, the active sites on N-GLPs 750_6_ contained intensive electrostatic attraction with BPA at higher temperatures [47]. Overall, the increase in temperature enhanced the interaction between BPA and N-GLPs 750_6_ and modified the adsorption condition. Considering removal efficiency and economic feasibility, we chose 50 °C as the ideal adsorption temperature.

#### 2.2.4. BPA Adsorption Isotherms and Thermodynamics Studies

It is important to identify and understand the adsorption mechanisms and the interactions between BPA and the adsorbents through equilibrium adsorption isotherms [50]. The adsorption isotherms of BPA on N-GLPs 750_6_ at different temperatures are shown in Figure S1. The Langmuir and Freundlich isotherms were utilized to analyze the equilibrium adsorption data. The equations are expressed as follows [42,51,52,53]:

(1) $$\mathrm{Langmuir}\;\mathrm{isotherm}:\;q_{e}=\frac{q_{\max}K_{L}c_{e}}{\left(1+K_{L}c_{e}\right)}$$

(2) $$\mathrm{Freundlich}\;\mathrm{isotherm}:\;q_{e}=K_{F}c_{e}^{1/n}$$

where *q_e_* is the equilibrium adsorption concentration of BPA (mg/g); *c_e_* is the equilibrium BPA concentration in aqueous solution (mg/L); *q_max_* is the maximum adsorption capacity (mg/g); *K_L_* is the Langmuir isotherm constant (L/mg); and *K_F_* and *n* are the Freundlich isotherm constant and adsorption capacity parameter, respectively.

Table S2 shows the values of *K_L_*, *q_m_*, *K_F_*, and *n* and the correlation coefficients for Langmuir and Freundlich. Compared with the Freundlich model (0.9580–0.9376), the Langmuir model (0.9982–0.9922) contained much better correlation coefficients, indicating that the adsorption of BPA on N-GLPs 750_6_ was inclined to monolayer adsorption on homogeneous adsorbent surfaces. Furthermore, with rising temperature, the increase in *q_m_* demonstrated that adsorption was facilitated.

The thermodynamic parameters were next evaluated by the following equations to confirm the conditions of the adsorption process [52,53]:

(3) $$K_{C}=\frac{q_{e}}{c_{e}}$$

(4) $$\Delta G{}^{\circ}=-RT\ln K_{C}$$

(5) ΔG°=ΔH°−TΔS°

(6) $$\ln K_{C}=-\frac{\Delta H{}^{\circ}}{RT}+\frac{\Delta S{}^{\circ}}{R}$$

where *K_C_* is the adsorption equilibrium constant, calculated by *q_e_* and *c_e_* at initial concentration = 10 mg/L; *R* is the gas constant (8.314 J∙mol^−1^ K^−1^); and *T* is the temperature in Kelvin.

As shown in Table S3, the negative values of ΔG° (−0.97 to −7.13 KJ/mol) suggested a spontaneous adsorption process, and the decrease in values with increasing temperature indicated that adsorption was promoted at high temperatures. The positive value of ΔH° (42.01 KJ/mol) also suggested that the adsorption process was endothermic, which led to the increase in adsorption capacity at higher temperatures. Moreover, the positive value of ΔS° (150.41 J∙mol^−1^∙K^−1^) showed an increase in the number of species and randomness at the N-GLPs 750_6_/water interface [54].

#### 2.2.5. Adsorption Mechanism

**Figure 13:**
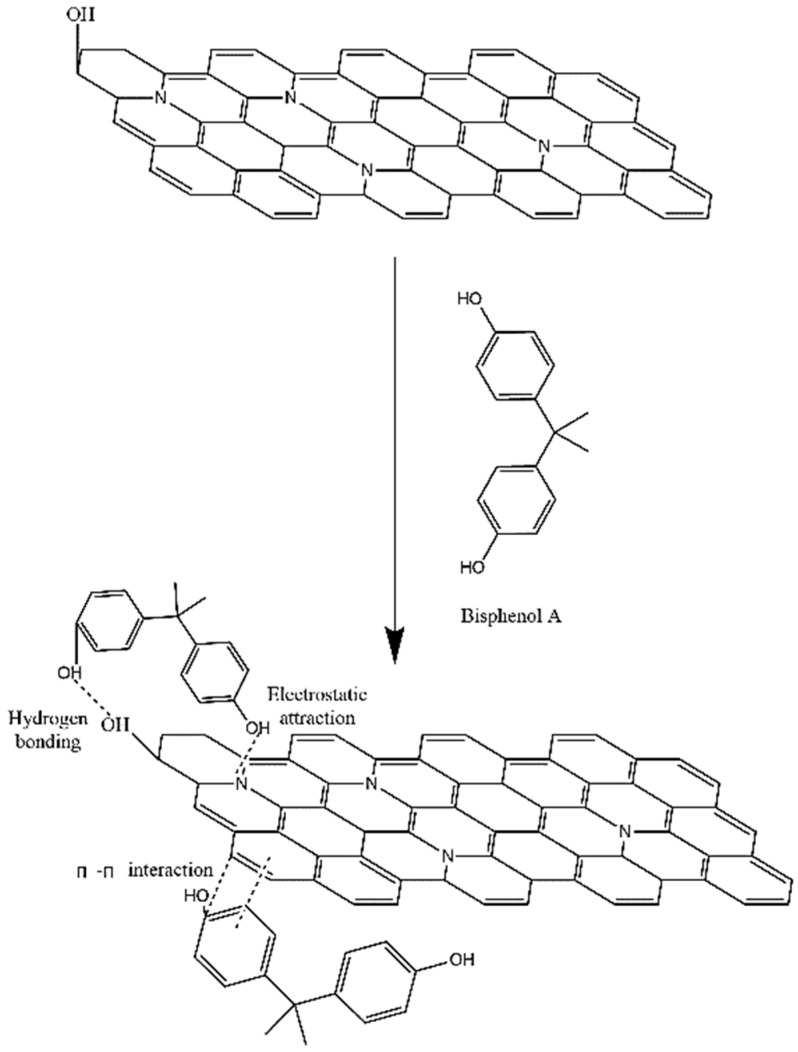
Schematic of adsorption interaction of bisphenol A (BPA) onto N-GLPs 750_6_.

The adsorption mechanism of BPA onto N-GLPs 750_6_ was further clarified with the structural analyses of N-GLPs 750_6_ and adsorption experiment. As shown in Figure 13, with the structural features of nitrogen-doped graphene-like plates and the adsorption isotherms, the following three main interactions could be related to the adsorption of BPA on N-GLPs 750_6_: (1) The strong electrostatic interaction between BPA and the N-GLPs 750_6_. Electrostatic attraction significantly promoted the adsorption process [55]. When interacting with negatively charged BPA in aqueous solution, the N-GLPs 750_6_ with positive charge possessed enhanced electrostatic attraction for removing pollutants, resulting in higher removal efficiency. (2) The hydrogen bonding between the oxygen-containing groups contained in both BPA and N-GLPs 750_6_. With the FT-IR result (Figure 7d), some oxygen-containing groups, such as hydroxyl groups, remained in N-GLPs 750_6_. In aqueous solution, the hydroxyl groups of BPA and adsorbent will form hydrogen bonds to increase the adsorption ability. (3) The π-π interaction that might exist between the N-GLPs 750_6_ planes and the benzene rings of BPA. N-GLPs 750_6_ possessed structural units that were similar to benzene rings through calcination and nitrogen doping. Therefore, π-π electron coupling might exist between the π electrons of benzene rings on BPA and N-GLPs 750_6_, thus forming a π-π interaction [56]. With all of these strong interactions, N-GLPs 750_6_ obtained a great adsorption capacity of BPA.

#### 2.2.6. Desorption and Regeneration of N-GLPs 750_6_

**Figure 14:**
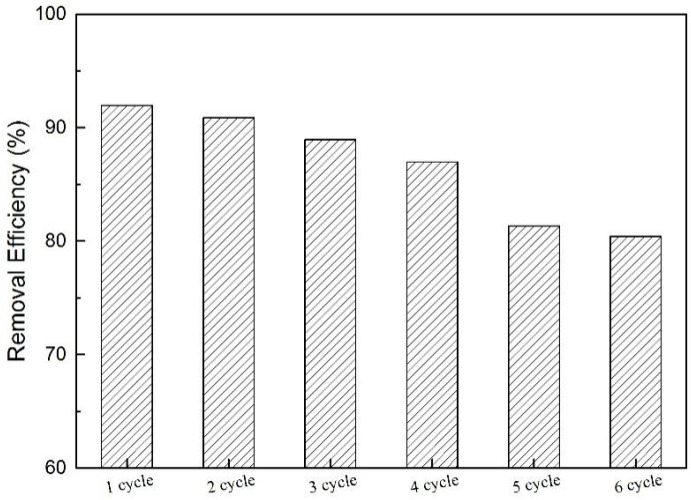
Recyclability of N-GLPs 750_6_ for six cycles.

In practical applications of pollutant removal, high adsorption capacity and good recyclability are requisite [57]. In this way, it is important to test the regeneration performance of N-GLPs 750_6_ in order to evaluate the economic feasibility in practical BPA removal applications. The regeneration of N-GLPs 750_6_ was determined using a simple solvent (ethyl/water: 4/1, *v*/*v*) washing method under ultrasound (US) irradiation [5]. The separation of N-GLPs 750_6_ and liquid phase was carried out by centrifuging at 9000 rpm for 15 min and then stoved in an oven at 80 °C for 30 min. The resulting N-GLPs 750_6_ sample was used again for the next recycling experiment. Adsorption-desorption experiments were repeated for six cycles in this study. Figure 14 shows that the removal efficiencies of BPA were decreased after each adsorption cycle. This phenomenon might have resulted from the occupation of the adsorption sites at the surface of N-GLPs 750_6_. After six adsorption-desorption cycles, the removal efficiency of BPA decreased from 92.0% to 80.4%, which is still an appropriate efficiency for industrial application. Therefore, according to the regeneration study, it was concluded that N-GLPs 750_6_ possess great regeneration ability and can be used in practical application for the treatment of BPA in wastewater.

A comparison was made of other carbonaceous adsorbents previously reported in the literature (Table 3) [58,59,60,61]. Although the removal efficiency of N-GLPs 750_6_ is narrowly lower than that of activated carbon, the preparation of N-GLPs 750_6_ is easier and more cost-effective because its raw material is green petroleum coke—a kind of potential pollutant and by-product. Besides, N-GLPs 750_6_ also contains great recyclability, which can further reduce the cost. The contact time for N-GLPs 750_6_ is also smaller than that of activated carbon. Considering the zeta potential of the adsorbents, the high affinity of N-GLPs 750_6_ to BPA is even more obvious. Therefore, N-GLPs 750_6_ is an excellent BPA absorbent in wastewater treatment.

## 3. Materials and Methods

### 3.1. Materials

Green petroleum coke with 93% carbon content was purchased from Chambroad Holding Group Co., Ltd. (Shandong, China). H_2_SO_4_ (98%) and NaOH with high chemical purity were purchased from Sinopharm Chemical Reagent Co., Ltd. (Shanghai, China). Melamine was purchased from Kemiou Chemical Reagent Co., Ltd. (Tianjin, China). Bisphenol A was purchased from Macklin Biochemical Co., Ltd. (Shanghai, China). Ethanol was purchased from Fuyu Fine Chemical Co., Ltd. (Tianjin, China). The latter three chemicals were all of analytical grade. Deionized water (resistivity of 18.2 MΩ cm^−1^) was achieved using an ultrapure water purifier system (Millipore, Billerica, MA, USA). All chemicals and materials were used without further purification.

### 3.2. Characterization

SEM (JSM-6700F, JEOL, Tokyo, Japan) was used to analyze the structural features and surface morphologies of the green petroleum coke, petroleum coke, and nitrogen-doped graphene-like plates. The acceleration voltage of SEM was 3.0 KV, and the mode of operation was SE. An energy-dispersive X-ray spectroscopy (EDX) instrument was attached to the JSM-6700F to investigate chemical composition. XRD was conducted on a diffractometer (Rigaku D-Max 2200, Tokyo, Japan) to characterize the structural features of samples at a scanning rate of 2°/min in 2θ range of 10–80°. The XRD data was analyzed by MDI Jade 6. FT-IR (Vector 22, Bruker AXS, Co., Ltd., Karlsruhe, Germany) of the samples was performed using KBr pellets in reflectance mode from 400 to 4000 cm^−1^ with a resolution of 2 cm^−1^ to examine the functional groups. A surface area and porosity analyzer (ASAP, 2020 HD88, Micromeritics, Norcross, GA, USA) was conducted to analyze the pore structure of samples. The BET specific surface area was calculated with multipoint adsorption data in relative pressure (P/P_0_) between 0.05 and 0.1, and the V_p_ was obtained at P/P_0_ = 0.998. XPS was performed to determine the elementary characteristics on the X-ray photoelectron spectrometer (Thermo ESCALAB 250XI, Thermo Fisher Scientific, Waltham, MA, USA). The source gun type was Al K alpha, and the pass energy was 100.0 eV for the XPS survey spectra and 30.0 eV for the C (1s), O (1s), N (1s) and S (2p) spectra. The XPS data was analyzed by XPS Peak 4.1. The zeta potential of the samples was measured by a ZetaPALS (Zetasizer NanoZS, Malvern, UK). All the figures were drawn by Origin 9.1 and Photoshop 2018.

### 3.3. Preparation of Petroleum Coke

Green petroleum coke was pulverized by a high-speed pulverizer and passed through a 200-mesh screen to prepare green petroleum coke powder within 75 μm of granularity. Green petroleum coke powder in a porcelain boat with a lid was then placed into a corundum tube with a flow of nitrogen atmosphere and heated to 200 °C at a rate of 10 °C/min and directly to 800 °C at a rate of 5 °C/min in a tubular furnace [32]. After the temperature was maintained for 6 h, the furnace was slowly cooled to room temperature, and the petroleum coke was directly collected from the porcelain boat. Compared to green petroleum coke, obvious changes occurred in petroleum coke in terms of morphology, microstructure, and particle size (Figure S2a,b). EDX quantitative microanalysis indicated the presence of C, N, O, and S in the green petroleum coke (Figure S3a). After calcination, the elemental content of N and O decreased slightly in petroleum coke, while S disappeared in the EDX spectrum of petroleum coke (Figure S3b). Combining the XPS survey spectra and C(1s), O(1s), and S(2p) spectra of green petroleum coke and petroleum coke (Figure S4) as well as the XPS atomic concentration report (Table S4), the S in green petroleum coke was successfully removed.

### 3.4. Preparation of Nitrogen-Doped Graphene-Like Plates

Nitrogen-doped graphene-like plates were synthesized by calcining a mixture of petroleum coke and melamine powder in a tubular furnace. Melamine powder (2.0 g) was added to 40 mL ethanol. After 30 min of stirring at 70 rpm at 60 °C, petroleum coke powder (2.0 g) was continuously added into the mixture, with stirring maintained for an additional 30 min. The mixture was then stoved in an oven at 80 °C for 30 min. The obtained grey-color powder mixture was loaded on a porcelain boat and inserted into a quartz tube of a tubular furnace for thermal annealing [32,38,62]. The N-doped graphene-like plate samples were obtained by annealing the mixture at 350–750 °C for 2–6 h under N_2_ flow.

### 3.5. Adsorption Experiments

The adsorption experiment of bisphenol A was carried out with batch techniques. The adsorbent (7.0 g/L) was added into BPA solution (10–100 mg/L) and mixed uniformly by shaking at 150 rpm for 48 h at different temperatures (10–60 °C). NaOH and HCl were utilized to adjust the pH to 4–11. After the adsorption process, the mixture was centrifuged at 9500 rpm for 15 min to separate the supernatants. The concentration of BPA was measured with a UV/vis spectrometer (TU-1810 PC, Purkinje, Beijing, China) and calculated by absorbance at 276 nm. The removal efficiency (R) for BPA was calculated as follows:

R = (c_0_ − c_t_)/c_0_ × 100% (7)

where c_0_ and c_t_ (mg/L) are concentration of pollutants at initial and specific adsorption times, respectively. The removal amount (Q_t_) of pollutants by samples was calculated as follows:

Q_t_ = (c_0_ − c_t_)V/W (8)

where V (L) is the volume of the solution, and W (g) is the mass of the catalysts. The adsorption isotherms were used to analyze the adsorption mechanisms of BPA onto N-doped graphene-like plates. The desorption and regeneration of the N-GLPs were also examined to figure out the recycling ability.

## 4. Conclusions

In summary, N-GLP samples were successfully synthesized via nitrogen doping and thermal annealing, which offered a potential low-cost and efficient adsorbent to treat BPA in aqueous solution. Green petroleum coke was used as a raw material due to advantages such as its abundance as waste in the oil-refining process and inexpensiveness. N-GLPs 750_6_ obtained by calcinating melamine and petroleum coke at 750 °C for 6 h exhibited a graphene-like structure according to SEM images and XRD analysis. Moreover, FT-IR analysis and XPS analysis of N-GLPs 750_6_ confirmed successful nitrogen doping. BET analysis demonstrated that nitrogen doping and calcination could increase the specific surface area and pore volume of the adsorbent in order to augment adsorption capacity. The zeta potentials of adsorbate and adsorbent indicated strong electrostatic attraction between positive N-GLPs 750_6_ and negative BPA, which was the main interaction enhancing the adsorption of more BPA onto the surface of N-GLPs 750_6_. Batch adsorption experiments revealed that the removal of BPA depended on contact time, initial pH, and temperature. Furthermore, adsorption experiments showed that N-GLPs 750_6_ possessed high adsorption capacity for BPA via electrostatic attraction, hydrogen bonding, and π-π interaction. Thermodynamic studies also revealed that the adsorption process was feasible, spontaneous, endothermic, and controlled by a physisorption process. Therefore, it is suggested that N-GLPs 750_6_ is a promising adsorbent to remove BPA in wastewater.

## Figures and Tables

**Table 1:** Surface area and porous volume of the green petroleum coke and N-doped samples.

Sample	S_BET_ (m^2^/g)	V_p_ (cm^3^/g)
Green petroleum coke	8.0	0.013
N-GLPs 350_2_	35.9	0.029
N-GLPs 550_2_	44.8	0.040
N-GLPs 750_2_	65.4	0.055

**Table 2:** Surface area and porous volume of the green petroleum coke and N-doped samples.

Sample	S_BET_ (m^2^/g)	V_p_ (cm^3^/g)
Green petroleum coke	8.0	0.013
N-GLPs 750_2_	65.4	0.055
N-GLPs 750_4_	76.9	0.068
N-GLPs 750_6_	96.6	0.082

**Table 3:** Adsorption capacity of BPA by N-GLPs 750_6_ in comparison to other literature values.

Adsorbent	Pollutant	pH	Contact Time (h)	C_0_ (mg/L)	Removal Efficiency (%)	Reference
Activated carbon	BPA	-	60	10	94.4	[58]
Porous carbon	BPA	-	24	5	90.0	[59]
CNTs	BPA	6.0	24	10	71.8	[60]
Nitrogen-doped graphene	BPA	6.0	12	10	90.5	[61]
N-GLPs 750_6_	BPA	6.0	48	10	92.0	This Work
N-GLPs 750_6_	BPA	6.0	48	5	98.3	This Work

## Supplementary Materials

The following are available online. Figure S1: Adsorption isotherms of BPA on N-GLPs 750_6_ (experimental conditions: adsorbent dose = 7.0 g/L, initial concentration = 10–200 mg/L, contact time = 48 h, pH = 6.0, T = 10–50 °C); Figure S2: SEM images of (a) green petroleum coke and (b) petroleum coke; Figure S3: EDX analysis of (a) green petroleum coke and (b) petroleum coke; Figure S4: XPS survey (1) and high-resolution C 1 s (2), O 1 s (3), S 2p (4) spectra of (a) green petroleum coke and (b) petroleum coke; Table S1: Pseudo-first-order and pseudo-second-order kinetic parameters for the adsorption of BPA onto N-GLPs 750_6_ at 303 K; Table S2: Langmuir and Freundlich adsorption isotherm parameters for the adsorption of BPA onto N-GLPs 750_6_; Table S3: Thermodynamic parameters for the adsorption of BPA onto N-GLPs 750_6_; Table S4: XPS atomic concentration report of green petroleum coke and petroleum coke.
