# Supplementary material for: Efficient Removal of Bisphenol A Using Nitrogen-Doped Graphene-Like Plates from Green Petroleum Coke
Source: Molecules. 2020 Aug 3;25(15):3543. doi: 10.3390/molecules25153543 (PMC7435634; doi:10.3390/molecules25153543)
Supplement: Supplementary file 1 [file molecules-25-03543-s001.pdf]

Supporting Information

Efficient Removal of Bisphenol A Using Nitrogen-doped Graphene-like Plates from Green Petroleum Coke

Zhipeng Liu[1], Quanyong Wang[2], Bei Zhang[1], Tao Wu[3,*], and Yujiang Li[1,*]

[1] Shandong Provincial Research Center for Water Pollution Control, School of Environmental Science and Engineering, Shandong University, Jinan 250100, PR China; 201812787@mail.sdu.edu.cn (Z.L.); zhangbeisdu@163.com (B.Z.)

[2] China Urban Construction Design and Research Institute Co., Ltd., Jinan 250101, PR China; hkyys@vip.sina.com (Q.W.);

[3] Key Laboratory of Colloid and Interface Science of Education Ministry, Shandong University, Jinan 250100, PR China

* Correspondence: wutao@sdu.edu.cn (T.W.), +86-15853196759; yujiang@sdu.edu.cn (Y.L.), +86-15953179619;

Table S1. Pseudo-first-order and pseudo-second-order kinetic parameters for the adsorption of BPA onto N-GLPs 750₆ at 303 K.

C_0 (mg/L)	$q_{e,exp}$ (mg/g)	Pseudo-first-order			Pseudo-second-order		
		$q_{e1,cal}$ (mg/g)	$k_1 \times 10^3$ (min^{-1})	R^2	$q_{e2,cal}$ (mg/g)	$k_2 \times 10^2$ ($g \cdot mg^{-1}\ min^{-1}$)	R^2
10	1.16	0.63	1.7	0.9152	1.17	1.0	0.9993
20	1.87	0.71	1.9	0.8343	1.89	1.0	0.9998
50	2.31	1.01	2.0	0.9103	2.34	0.8	1
100	4.42	1.54	1.9	0.8347	4.45	0.6	0.9999
200	5.89	1.96	2.2	0.872	5.95	0.5	1

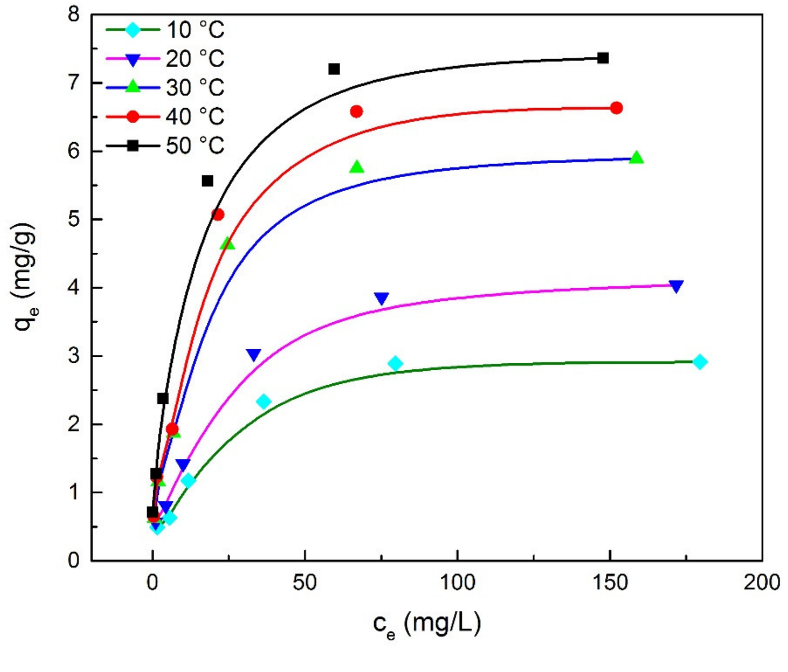

Figure S1. Adsorption isotherms of BPA on N-GLPs 750_6 (experimental conditions: adsorbent dose = 7.0 g/L, initial concentration = 10-200 mg/L, contact time = 48 h, pH = 6.0, T = 10-50 °C).

Table S2. Langmuir and Freundlich adsorption isotherm parameters for the adsorption of BPA onto N-GLPs 750_6.

Temperature(K)	Langmuir isotherm			Freundlich isotherm		
	q_{max} (mg/g)	K_L (L/mg)	R^2	K_F	n	R^2
283	3.21	0.064	0.9922	13.54	0.46	0.9376
293	4.42	0.067	0.9936	4.62	0.45	0.9565
303	6.28	0.108	0.9962	1.60	0.45	0.9568
313	7.06	0.117	0.9936	1.08	0.44	0.9580
323	7.61	0.211	0.9982	4.12	0.34	0.9553

Table S3. Thermodynamic parameters for the adsorption of BPA onto N-GLPs 750_6.

Temperature (K)	$\Delta G°$(KJ·mol^{-1})	$\Delta H°$(KJ·mol^{-1})	$\Delta S°$(J·mol^{-1}·K^{-1})
283	-0.97	42.01	150.41
293	-1.40		
303	-4.02		
313	-4.30		
323	-7.13		

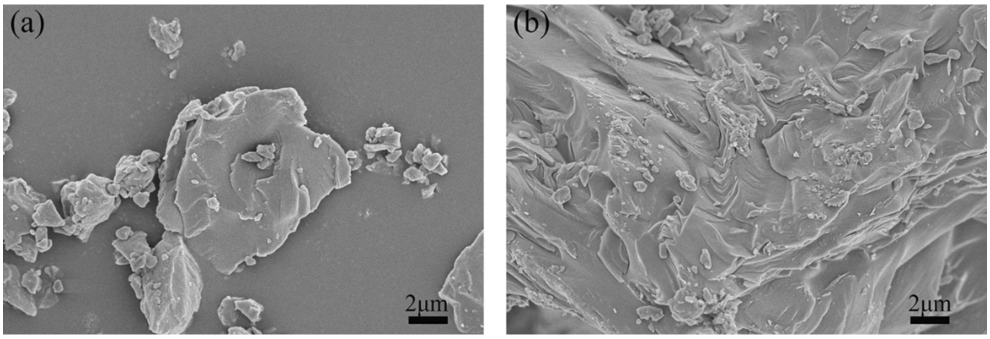

Figure S2. SEM images of (a) green petroleum coke and (b) petroleum coke.

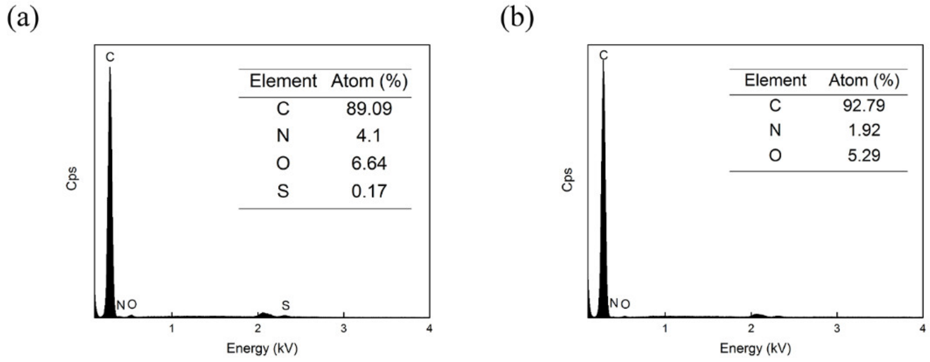

Figure S3. EDX analysis of (a) green petroleum coke and (b) petroleum coke.

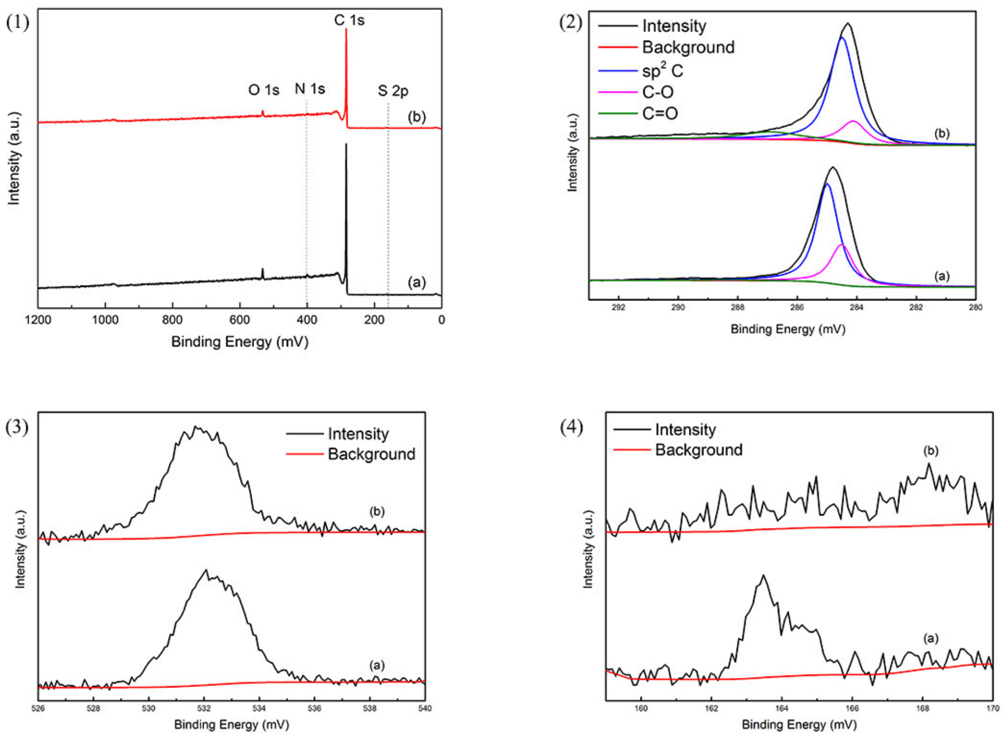

Figure S4. XPS Survey (1) and high-resolution C 1 s (2), O 1 s (3), S 2p (4) spectra of (a) green petroleum coke and (b) petroleum coke.

Table S4. XPS atomic concentration report of green petroleum coke and petroleum coke.

Element	Green petroleum coke (%)	Petroleum coke (%)
C	93.12	94.40
N	2.25	1.56
O	4.34	4.04
S	0.29	0
